# Effects of an electrolyte, energy, and osmolyte compound and heat stress on productivity in early-lactation Holstein cows

**DOI:** 10.3168/jdsc.2025-0868

**Published:** 2025-12-18

**Authors:** D. Onan-Martinez, C. Nelson, F.T. Saputra, A. Fraz, C. Law, J. Lance, H. Olmo, I.M. Toledo, L.T. Casarotto, Y. Wen, N.C. Upah, G.E. Dahl

**Affiliations:** 1 Department of Animal Sciences, University of Florida, Gainesville, FL 32608; 2 TechMix LLC, Stewart, MN 55385

## Abstract

Heat stress affects milk production and intake of dairy cattle. Strategies to mitigate the effects of heat stress suggest dietary supplements including electrolyte, energy, and osmolyte compounds (such as Bovine BlueLite, TechMix, Stewart, MN, which is a blend of these compounds), but direct and long-term evaluation of this supplement in lactating cows is needed. We hypothesized that the supplement would improve productivity of dairy cows (∼52 DIM) under heat stress. Cows (n = 52) were enrolled into a 4-wk 2 × 2 factorial arrangement as follows: (1) heat stress control (HT-CON, n = 13), (2) heat stress plus supplement (HT-SUP, n = 13), (3) cooling control (CL-CON, n = 13), and (4) cooling plus supplement (CL-SUP, n = 13). Cows were housed in a freestall barn. Supplement treatment was top-dressed on TMR (56.5 g/cow in the morning and 56.5 g/cow in the afternoon feeding). Evaporative cooling was provided to cows in the cooling groups, whereas heat-stressed cows only had shade. Milk yield, DMI, rectal temperature (RT), and respiration rate (RR) of individual cows were recorded during the study. Furthermore, milk contents of fat, protein, and lactose were measured weekly. Heat stress increased RR and RT relative to the cooling groups. Heat stress reduced DMI (HT = 20.3, CL = 24.5 ± 0.4 kg/d; ± SE), and supplementation reversed some of the effects in heat-stressed cows (HT-SUP = 20.9, HT-CON = 19.6 ± 0.5 kg/d) but did not affect cooled cows. The ECM yield was also decreased by heat stress (HT = 38.1, CL = 42.6 ± 0.8 kg/d), and supplementation partially reversed that effect in HT-SUP relative to HT-CON (HT-SUP = 39.6, HT-CON = 36.5 ± 1.1 kg/d). No main effects were observed for milk components, cortisol, or heat shock protein plasma concentration. Results suggest that supplementation partially mitigated the effects of heat stress on DMI and ECM in lactating dairy cows.

## Highlights

• Heat stress negatively affected feed intake and milk production in early-lactation dairy cows.
• The supplement (electrolyte and energy compound) partially mitigated the negative effects of heat stress on energy-corrected milk (ECM) in the last week of supplementation.
• Milk percentages of fat, protein, and lactose were unaltered by the effects of environment or supplement.
• Blood cortisol and heat shock protein concentration were unaltered by the effects of environment or supplement.

## Graphical Abstract

**Figure:**
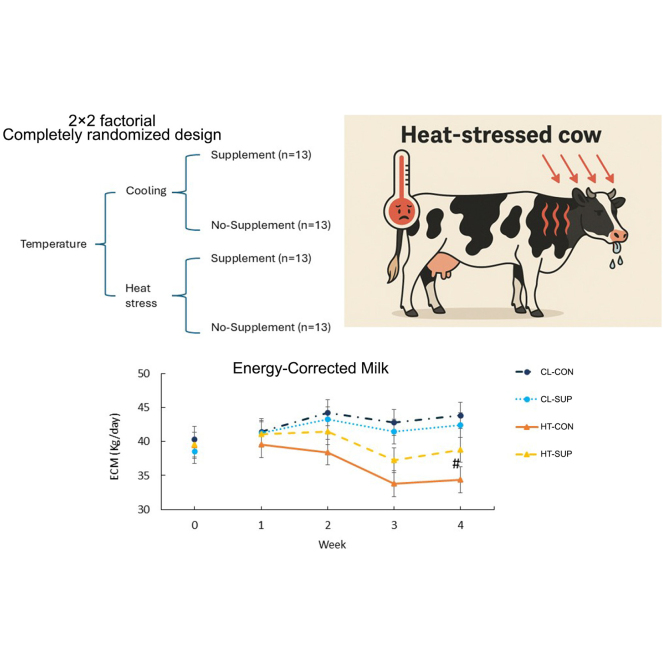
**Summary:**Heat stress impairs milk production and feed intake in dairy cows. This study tested whether a dietary supplement (containing electrolyte, osmolyte, and energy compounds) could help cows cope with heat stress. A total of 52 early-lactation cows were assigned to 1 of 4 groups for 4 weeks: (1) heat stress control (HT-CON), (2) heat stress plus supplement (HT-SUP), (3) cooling control (CL-CON), and (4) cooling plus supplement (CL-SUP). Cows received the supplement top-dressed on their feed. Cooling cows had access to an evaporative cooling system, whereas heat-stressed cows only had shade. We measured milk production, feed intake, body temperature, and respiration rate. Heat stress increased body temperature and breathing, and reduced feed intake and milk yield, whereas the supplement partially helped reduce these effects in heat-stressed cows but had no effect on cows with automated cooling. Milk composition and blood stress markers were not significantly affected.

Heat stress is a major issue for animal production, especially in tropical and subtropical areas, and is increasing due to global warming (Chen et al., 2021). Homeotherms need to release metabolic heat to the environment to maintain normal body core temperature. When heat production exceeds heat dissipation, the ability to release heat is limited, leading to heat stress (**HS**), which affects metabolism and performance (Collier et al., 2006). Humidity is a major contributor to HS; therefore, the temperature-humidity index (**THI**) has been considered a more accurate tool than temperature alone to determine the HS load of livestock in a confinement setting (Collier et al., 2006). In dairy cattle, the threshold THI associated with production losses is as low as 64 (Ji et al., 2020). All southern regions of the United States have high average temperatures, and in Florida, THI levels easily surpass the 64 THI threshold even during the coldest months of the year.

Negative effects from exposure of dairy cattle to HS include reduced DMI, lower milk yield, body condition loss, and reduced feed efficiency, resulting in important economic losses (Brown-Brandl, 2018). Reduction of DMI is an adaptive mechanism to minimize heat production from feed fermentation and metabolism (Chang-Fung-Martel et al., 2021). Moreover, HS induces reduction of peristalsis in the digestive system, which delays digestive function and feed metabolism and, thus, feed intake (Chen et al., 2021). As a result, HS is detrimental to milk yield, representing a major issue for dairy producers. Indeed, milk production losses can exceed 30% under severe HS, and milk yield declines an average of 0.2 kg for every THI-unit increment once the threshold is surpassed (West, 2003).

In an attempt to mitigate HS, cooling systems that include shade, fans, and soakers have been deployed heavily in the dairy industry. These systems prevent reductions in cows' feed intake and milk yield, especially when THI exceeds 68 (Chang-Fung-Martel et al., 2021). Additionally, dietary strategies have been employed to mitigate the negative influence of HS, with differing levels of effectiveness. These approaches are oriented to support water balance, ensure adequate intake of nutrients and electrolytes, and address specific nutritional requirements during periods of HS, such as increased needs for vitamins and minerals (Renaudeau et al., 2012). Supplementation with products containing electrolytes, osmolytes, and energetic compounds have shown to have potential metabolic support in lactating cattle under HS (Al-Qaisi et al., 2020a). Therefore, we hypothesized that supplementation of electrolyte and energetic compounds (BlueLite pellets, TechMix, Stewart, MN) during extended HS of early-lactation cows will reduce the negative effects on productive parameters and circulating indicators of HS.

Our study was conducted during the summer of 2022 at the University of Florida Dairy Unit with approval of all procedures by the Institutional Animal Care and Use Committee. A total of 52 lactating Holstein cows with an average of 52 DIM (SD = 28 d) were enrolled in a 2 × 2 factorial arrangement in a completely randomized design, yielding 4 groups of 13 cows each, with individual cow as the experimental unit. Each cow was randomly assigned to one of the combinations of environment (heat stress treatment, **HT**, vs. cooling treatment, **CL**) and supplement (supplementation group, **SUP**, vs. unsupplemented control group, **CON**) treatments as the main factors, as follows: (1) heat stress control (HT-CON), (2) heat stress plus supplement (HT-SUP), (3) cooling control (CL-CON), and (4) cooling plus supplement (CL-SUP), during a period of 28 d, balancing each group based on their PTA for milk yield, DIM, and parity. The CL treatment consisted of shade, fans (located ∼3 m above the line of beds and activated automatically at ∼20°C; wind speed from fans was ∼3.6 km/h), and soakers (located above the feed line and activated automatically at ∼20°C for cycles of 30 s on and 5 min off and 1 min on and 5 min off after ∼24°C), whereas the HT treatment was only shade. The supplement was offered top-dressed on the TMR at a rate of 113 g/d (per manufacturer recommendation) divided into 56.5 g at morning (0800 h) and afternoon (1300 h) feedings for the SUP cows, whereas the CON cows did not receive any supplementation or carrier. The basal TMR composition was as follows (%, DM basis): corn silage, 37.3; oat silage, 3.9; ground corn grain, 23.2; soybean meal, 9.8; AminoPlus (bypass soybean meal, Ag Processing Inc., Omaha, NE), 6.0; fresh cow minerals, 5.5; Nurisol (calcium salt of long-chain fatty acids, Global Agri-Trade, Rancho, CA), 1.0; citrus pulp, 9.4; and molasses, 1.9. The TMR had the following calculated nutrient analysis (DM basis): crude protein, 15.71%; ME allowable milk 43.5 kg/d; amylase-treated NDF on an OM basis, 21.29%; starch, 31.47%; ether extract, 3.62%; and DCAD, 226.99 mEq/kg. Additionally, the ingredients of the supplement include electrolytes (Na, K, Cl, S, Mg, Ca), vitamins (B, A, D, E), energetic compounds (dextrose and sucrose), and an osmolyte (betaine). The calculated analysis of the supplement shows DCAD of 58.36 mEq/kg. The study was conducted during the summer when THI was on average 77.2 ± 1.3 (± SD), a THI that induces HS in cows in the absence of fans and soakers. Cows were housed in a freestall barn equipped with automatic individually activated gates for each feeder (Calan Gate System, American Calan) to measure individual feed intake. Cows were fed ad libitum, calculating their individual feed intake by offering feed in the morning and afternoon and measuring residual feed after 24 h relative to the morning feeding. Milk yield was recorded throughout the study period after each morning and evening milking. In addition, milk samples were collected weekly from consecutive morning and evening milkings from each cow, and milk component analysis for fat, protein, and lactose, as well as MUN and SCC, was subsequently performed at Southeast Milk Inc. Laboratory (Belleview, FL). Milk and component values were then converted to ECM using the following formula: ECM, kg/d = [(0.3246 × milk yield) + (12.86 × fat yield) + (7.04 × protein yield)]; and SCC were transformed to SCS as follows: SCS = [log_10_(SCC/12.5)]/log_10_(2).

Rectal temperature (**RT**) was measured using a digital thermometer (Pavia Rectal Temp, Pavia Sales Group Inc., Minnetonka, MN), and respiration rate (**RR**) was measured by counting thoracic movements for 1 min. Vitals were assessed every 2 d in all cows at 1400 h to confirm the effect of HS.

In addition, a single blood sample was collected from a subset of 40 cows (10 cows randomly selected per treatment group) via coccygeal venipuncture on d 0, 7, 14, and 28 relative to the beginning of the treatments, with all collections performed at 0600 h, using heparinized evacuated tubes (BD Vacutainer, Franklin Lakes, NJ). After collection, blood samples were centrifuged at 1,006 × *g* for 15 min at 4°C, and plasma was separated and stored at −80°C until analysis of cortisol and heat shock protein (**HSP**). Cortisol concentration in samples was measured using an ELISA commercial kit with intra- and interassay coefficients of variation of 8.33% and 8.78%, respectively (K003-H1/H5, DetectX Cortisol, Arbor Assays, Ann Arbor, MI); and HSP70 concentration in samples was measured using an ELISA commercial kit for cattle with an intra- and interassay coefficients of variation of 4.82% and 18.72%, respectively (SEA873Mi, Cloud-Clone Corp., Katy, TX).

Data collected were analyzed using the PROC MIXED procedure of SAS (version 9.4, SAS Institute Inc., Cary, NC). The model considers fixed effects of environment (HT vs. CL), supplement (SUP vs. CON), time, environment by supplement interaction, and environment by supplement by time interaction, and the random effect of cow nested withing treatment. The PTA for milk yield was included as a covariate in the analysis for DMI, milk yield, and milk components; however, it was not significant, and therefore it was removed. In addition, values of 1 wk before starting treatments were included as covariates for DMI, milk yield, milk components, cortisol, and HSP. Post hoc pairwise comparisons were performed using the Tukey–Kramer adjustment. Finally, residuals were tested for normality using the plots = studentpanel option in SAS. Statistical significance was considered when *P* < 0.05 and tendency when *P* < 0.10. Data are presented as LSM with SEM.

Respiration rate showed an environment by supplement interaction (*P* = 0.04), where SUP reduced RR in cows under HT but did not affect RR of cows under CL (Table 1). Moreover, main effects of environment showed that RR was higher for HT cows relative to CL cows (92.6 ± 1.0 vs. 82.2 ± 1.0 breaths/min; *P* < 0.01) whereas main effect of supplement showed that SUP tended to reduce RR relative to CON cows (86.0 ± 1.0 vs. 88.9 ± 1.0 breaths/min; *P* = 0.06). A similar pattern was observed for RT (°C), where a significant environment by supplement interaction was observed (*P* = 0.01); SUP reduced RT in cows under HT but did not affect RT in cows under CL (Table 1). An environment main effect was detected, where HT cows had higher RT than CL cows (39.83 ± 0.07 vs. 38.80 ± 0.07°C; *P* < 0.01), but there was no main effect of supplement (39.30 ± 0.07 vs. 39.34 ± 0.07°C; *P* = 0.74).

**Table 1:** Effects of environment (CL or HT) and supplementation (SUP or CON) on respiration rate, rectal temperature (HT-CON, n = 13; HT-SUP, n = 13; CL-CON, n = 13; and CL-SUP, n = 13), and blood cortisol and blood HSP (CL-CON, n = 8; CL-SUP, n = 7; HT-CON, n = 10; and HT-SUP n = 9), including *P*-values for main effects of environment, supplement, and environment by supplement interaction

Item	Treatment				SEM	*P-*value		
	CL-CON	CL-SUP	HT-CON	HT-SUP		Enviro^1^	Supple^2^	Enviro × Supple
Respiration rate, breaths/min	82.1^b^	82.4^b^	95.6^a^	89.7^c^	1.4	<0.01	0.06	0.04
Rectal temperature, °C	38.70^b^	38.92^b^	39.98^a^	39.69^c^	0.10	<0.01	0.74	0.01
Cortisol,^3^ ng/mL	10.99	8.42	8.67	11.01	1.07	0.90	0.91	0.03
HSP, ng/mL	74.75	76.40	77.70	75.76	10.7	0.91	0.99	0.86

a–c LSM values in the same row with different superscripts are different (*P* < 0.05). Comparisons were performed following Tukey–Kramer adjustment.

1 Main effect of environment (CL or HT).

2 Main effect of supplement (SUP or CON).

3 Despite the environment by supplement interaction, post hoc analyses indicated that after Tukey–Kramer adjustment of pairwise differences, treatment combinations were not different.

For DMI, a significant environment by supplement interaction was observed (*P* = 0.05) (Table 2), yet pairwise comparison after Tukey adjustment indicated that differences were driven by the main effect of environment, as HT cows had decreased DMI compared with CL cows (20.3 ± 0.4 vs. 24.5 ± 0.4 kg/d; *P* < 0.01), and no main effect of supplement was observed (22.5 ± 0.4 vs. 22.3 ± 0.4 kg/d; *P* = 0.60).

**Table 2:** Effects of environment (CL or HT) and supplement (SUP or no SUP) on DMI, milk yield, ECM, milk component percentages, and milk component yield (HT-CON, n = 13; HT-SUP, n = 13; CL-CON, n = 13; and CL-SUP, n = 13), including *P*-values for main effects of environment, supplement, and environment by supplement interaction

Response	Treatment				SEM	*P-*value		
	CL-CON	CL-SUP	HT-CON	HT-SUP		Enviro^1^	Supple^2^	Enviro × Supple
DMI,^3^ kg/d	24.9	24.1	19.6	20.9	0.5	<0.01	0.60	0.05
Milk yield, kg/d	40.4	40.6	35.0	37.7	1.1	<0.01	0.21	0.26
ECM, kg/d	42.6	42.6	36.5	39.6	1.1	<0.01	0.15	0.17
Efficiency, milk yield/DMI	1.59	1.71	1.87	1.79	0.07	<0.01	0.73	0.12
Efficiency, ECM/DMI	1.70	1.79	1.91	1.89	0.06	0.01	0.65	0.36
Milk components, %								
Fat^3^	3.99	3.79	3.81	3.92	0.09	0.56	0.49	0.05
Protein	3.30	3.32	3.18	3.29	0.06	0.20	0.23	0.48
Lactose	4.85	4.84	4.93	4.85	0.05	0.41	0.56	0.52
MUN, ng/dL	13.9	14.1	14.6	14.6	0.38	0.09	0.75	0.73
SCS	3.39	3.36	2.75	2.54	0.28	<0.01	0.74	0.66
Milk component yields, kg/d								
Fat	1.63^a^	1.52^ab^	1.32^b^	1.47^ab^	0.06	<0.01	0.81	0.05
Protein	1.41	1.40	1.17	1.30	0.06	<0.01	0.27	0.17
Lactose	1.97	1.97	1.74	1.81	0.09	0.03	0.66	0.64

a,b LSM values in the same row with different superscripts differ (*P* < 0.05) for the environment × supplement interaction. Comparisons were performed following Tukey–Kramer adjustment.

1 Main effect of environment (CL or HT).

2 Main effect of supplement (SUP or CON).

3 Despite the environment by supplement interaction, post hoc analyses indicated that after Tukey adjustment of pairwise differences, treatment combinations were not different.

As expected, the main effect of environment was observed, where HT decreased milk yield relative to CL cows (36.4 ± 0.8 vs. 40.5 ± 0.8; *P* < 0.01). Supplement or the interaction were not significant (*P* = 0.21 and *P* = 0.26, respectively). Milk yield was converted to ECM, and cows under HT produced significatively less ECM than cows in the CL group (38.1 ± 1.8 vs. 42.6 ± 1.8; *P* < 0.01). Supplement and environment by supplement interaction were not significant (*P* = 0.15 and *P* = 0.17). A triple interaction (environment by supplement by week interaction) was observed for ECM (*P* = 0.02), where the slice by week function with Tukey adjustment showed that when comparing HT-SUP against HT-CON, the HT-SUP combination tended to have an increased ECM in wk 4 (*P* = 0.09) relative to HT-CON (Figure 1).

**Figure 1:**
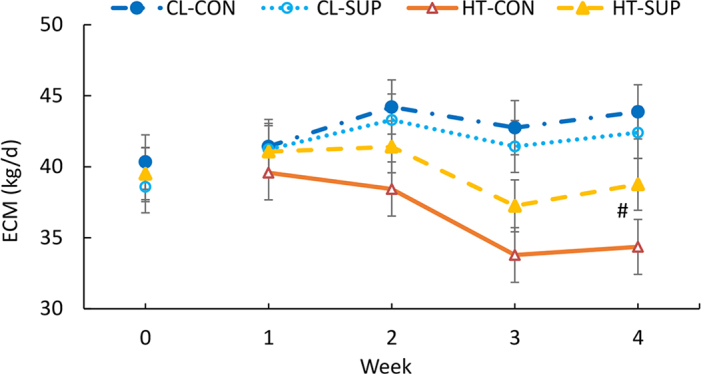
Energy-corrected milk of cows exposed to cooling system alone (CL-CON, solid circles with dash-dotted dark blue line), cooling system and supplement (CL-SUP, open circles with dotted light blue line), heat stress alone (HT-CON, open triangles with solid orange line), and heat stress and supplement (HT-SUP, solid triangles with dashed yellow line). HT-CON, n = 13; HT-SUP, n = 13; CL-CON, n = 13; and CL-SUP, n = 13. Effect of environment (*P* < 0.01), supplementation (*P* = 0.15), environment by supplement interaction (*P* = 0.17), and environment by supplement by time interaction (*P* = 0.02). Week 0 measurements were taken before starting treatments and were included in the model as covariate. The SLICE function of SAS (version 9.4, SAS Institute Inc., Cary, NC) was used to analyze individual week pairwise differences with Tukey adjustment. #*P* ≤ 0.10. Error bars represent SE.

Feed efficiency was calculated by dividing milk yield by DMI, where HT cows showed higher feed efficiency compared with CL (1.83 ± 0.05 vs. 1.65 ± 0.05; *P* < 0.01); however, supplement was not significant (*P* = 0.73). In addition, feed efficiency calculated with ECM was also increased by HT relative to CL (1.90 ± 0.04 vs. 1.74 ± 0.04; *P* = 0.01), nonetheless, supplement was not significant (*P* = 0.65). In both HT and CL groups, feed efficiency was calculated by milk yield and by ECM, and no environment by supplement interaction was detected (Table 2). Milk components, including the percentages of fat, protein, and lactose, were not affected by the main effects of environment nor supplement (*P* > 0.10); however, an environment by supplement interaction was found for fat percentage (*P* = 0.05; Table 2). Despite this interaction, pairwise comparisons adjusted by Tukey–Kramer did not reach statistical significance.

We found that HT tended to increase MUN (*P* = 0.09), whereas SCS was lower (*P* < 0.01) in HT cows relative to CL, and no supplement nor interaction effects were observed for MUN or SCS (Table 2). Milk component yields (kg/d) were lower in HT compared with CL (i.e., lactose, *P* < 0.01; protein, *P* < 0.01; and fat, *P* = 0.03), but no supplement effect was observed, and only fat yield showed an environment by supplement interaction (*P* = 0.05; Table 2). Post hoc comparisons using the Tukey–Kramer adjustment identified a significant difference only between CL-CON and HT-CON (*P* = 0.008).

Plasma cortisol and HSP concentrations from a subset of cows were evaluated. Initially, samples from 10 cows of each group were randomly selected for cortisol and HSP analysis; however, some samples were discarded due to hemolysis, resulting in sample numbers as follows: CL-CON, n = 8; CL-SUP, n = 7; HT-CON, n = 10; and HT-SUP, n = 9. In addition, values from d 0 were included in the model as covariate. Cortisol showed an environment by supplement interaction (*P* = 0.03); however, Tukey's adjusted pairwise comparisons showed no significant differences among main factor–level combinations. Additionally, we found no environment (*P* = 0.90) nor supplementation effects (*P* = 0.90; Table 1) for cortisol. We found that HSP was not influenced by environment (*P* = 0.91) or supplement (*P* = 0.99), nor was an interaction present (*P* = 0.86; Table 1).

Average THI in the barn during the experiment was 77.2 ± 1.3, which is expected to induce HS, especially in the absence of an effective evaporative cooling system. A THI of 64 is considered the borderline between thermoneutral zone and HS for lactating cows producing 31 kg of milk or more (Ji et al., 2020). Both RR and RT increased in HT cows due to an imbalance between heat production and dissipation, reflecting that cows carry more heat load compared with CL cows. However, the environment by supplement interaction for RR and RT shows that supplementation improved RR and RT in the HT cows. This effect may be attributed to enhanced fluid retention and heat exchange, likely driven by the role of betaine in maintaining intracellular fluid balance (Abhijith et al., 2024).

The DMI of cows under HT was reduced by 4.2 kg/d compared with CL cows. When under HS, the cooling center located in the hypothalamus signals the appetite center to decrease intake, resulting in lower heat production from fermentation (Albright and Alliston, 1971). Consequently, milk yield, ECM, and milk component yields in HT cows also decreased, due to both lower intake and negative effects of HS on mammary epithelial cells (i.e., increased apoptosis rate) due to HS (Tao et al., 2018), as shown in the HT cows, which produced 4.5 kg of ECM less than the CL group. Collier et al. (1982) showed that HS decreases sodium (Na) and potassium (K) concentrations in the rumen; moreover, sweating and panting induced by HS cause loss of electrolytes, further disrupting electrolyte balance. Indeed, supplementation of electrolyte compounds has been demonstrated to reduce negative effects of HS on milk production, as reported by West (2003). Betaine is an osmolyte compound that contributes to maintain intracellular electrolyte and fluid balance in many species, including cattle (Abhijith et al., 2024). BlueLite is a combination of energy, osmolyte, and electrolyte compounds, including betaine, K, and Na, and thus it is expected that its supplementation ameliorates HT effects, as in previous studies where individual components were tested (Collier et al., 1982; Abhijith et al., 2024). The present study clearly shows the negative effects of HT on DMI and ECM compared with CL cows. Although supplementation did not fully reverse the effects of HT to the same level of CL cows, supplementation did ameliorate a portion of the negative effects of HT on ECM in wk 4 of the experiment. The effect of supplementation on ECM in HT-SUP cows relative to HT-CON cows may be explained by the replacement of lost dietary electrolytes, the contribution of the supplement to increased DCAD, plus the positive effects of betaine, all of which may help maintain electrolyte balance. Al-Qaisi et al. (2020a) did not observe differences in DMI and milk yield with this supplement; however, in their trial cows were only exposed to a few days of HS. Conversely, in our experiment cows were exposed to HS for 4 wk, which allows for analysis of long-term effects of the supplement. Indeed, relative to HT-CON cows, HT-SUP cows benefited from supplementation as the trial advanced, which indicates that benefits of the supplement might increase as the HS insult lengthens, although supplementation does not allow for full replacement of active cooling conditions.

Feed efficiency is a key indicator of the effectiveness with which dairy cows convert feed into milk. We calculated efficiency based on milk yield and ECM and found an increase in feed efficiency in HT cows compared with CL, but supplementation did not show any significant effect. Our results match with those of a study by Hill and Wall (2017), where they collected a large data set from cows at different THI levels and found that, as THI increased, feed efficiency also increased. However, a meta-analysis by Chen et al. (2024) concluded that feed efficiency did not change due to HS. In our study, as opposed to Chen et al. (2024) but similar to Hill and Wall (2017), we enrolled early-lactation cows, which are known to mobilize body energy reserves to meet the increasing demand of milk production (Bernabucci et al., 2010). Consequently, in early lactation, a reduction in feed intake induces greater body energy mobilization to maintain milk yield, resulting in an apparent increase in feed efficiency. Moreover, the composition of the supplement includes energy components such as dextrose, sucrose, and fructose. However, the supplement was administered at a rate of 113 g/cow per day, which is insufficient to offset the reduction in TMR intake induced by HT (4.2 kg/d reduction), therefore not significantly altering feed efficiency.

The percentages of milk components (i.e., fat, protein, and lactose) were not affected by the main effects of environment nor supplement. Although an interaction was observed for milk fat, Tukey–Kramer adjusted pairwise comparisons did not reach statistical significance, suggesting high variability, likely due to sample sizes insufficient to detect differences in this variable. Studies evaluating milk component changes due to HS have yielded variable results. Al-Qaisi et al. (2020b) found an increase in milk fat percentage due to HS, whereas Bouraoui et al. (2002) reported decreased milk fat percentage during hotter months. Similarly, some studies indicate that milk protein percentage is reduced due to lower nutrient intake, thereby limiting milk protein synthesis under HS (Bernabucci et al., 2010; Al-Qaisi et al., 2020b), but, as with milk fat, differences were not observed in our study. In general, lactose percentage suffers little if any change with HS or other dietary manipulation, which is consistent with its function as the osmotic regulation of milk secretion (Cowley et al., 2015; Al-Qaisi et al., 2020b). No differences in milk components have consistently been found with electrolyte supplementation when under HS (West, 2003; Cabrera, 2014; Al-Qaisi et al., 2020a), which aligns with our findings. More research is needed to clarify the variability in fat and protein percentage under HS and supplementation with products with similar composition.

Milk fat yield also showed an environment by supplement interaction. Pairwise comparisons after Tukey adjustment only indicated differences between CL-CON and HT-CON, reinforcing the negative effects of HT. The lack of difference between HT-SUP cows and both groups of CL cows suggests that supplementation under HS conditions might help maintain fat yield similar to that of cows under cooling conditions, which also aligns with the observed improvement in ECM.

The HT groups tended to increase MUN, potentially due to lower DMI, which limits protein supply; additionally, studies have shown that plasma urea nitrogen also increases under HS, indicating muscle protein breakdown as a compensatory response to decreased protein intake (Bernabucci et al., 2010). Furthermore, in contrast to expected, HT decreased SCS relative CL. Different results are found on SCC and SCS across literature due to HS. Al-Qaisi et al. (2020a) found an increase in SCC, whereas Al-Qaisi et al. (2020b) did not observe such differences due to HS. Similar to ours, earlier research that included a large data set also found that HS decreased SCS (Smith et al., 2013). More research is needed to clarify the effects of HS on SCC. Additionally, as in the Al-Qaisi et al. (2020a) study, no effect of supplementation was detected in our study.

Cortisol showed an environment by supplement interaction (Table 1); however, Tukey adjusted pairwise comparison showed no significant differences, which might suggest high variability. Studies in beef (Kim et al., 2022) and dairy cattle (Marins et al., 2021) have shown that acute HS increases plasma cortisol concentrations, and animals exhibit a decrease in cortisol patterns back toward normal as they adapt to chronic HS exposure, which might contribute to explaining the lack of differences in cortisol, as we sampled at d 7 relative to beginning of treatments, when cortisol levels might have gone down after adaptation. Similarly, HSP plasma concentration is highly variable, especially in dairy breeds and within individuals of a homogeneous breed (Rakib et al., 2024). Responses of HSP to HS stimulus in in vitro studies with cell cultures seem to be more evident than in in vivo studies (Collier et al., 2018). In the present study, no main effects of environment, supplement, or their interactions were observed for HSP. The lack of difference in HSP plasma concentrations might be explained by the fact that even when CL cows are provided with evaporative cooling system, they still express signs of HS due to challenging environmental conditions during the summer in Florida. Indeed, the CL group showed elevated RR in the present study (i.e., ∼82.2 breaths/min) relative to a normal RR for lactating cows (i.e., 60 breaths/min), meaning that all cows were experiencing some level of HS, likely including before the initiation of treatments, which could have triggered some adaptative physiological changes that masked any increase in plasma cortisol and HSP during our study.

In the present study, environmental conditions were sufficient to induce HS in the absence of active cooling, as shown by the responses of RT and RR of HT cows, confirming the effectiveness of our HT model. Intake and milk yield were heavily compromised due to HT, and the supplement partially reversed those effects of HT. Moreover, the effects of the supplement become more evident after supplementation during several weeks of HT. Although HT had no effect on milk components overall, supplementation appeared to rescue some effects of HT on metabolism and allow for greater milk fat percentage. Furthermore, cortisol and HSP plasma concentrations were not affected by the main effects of environment or supplement in our experiment; however, caution is warranted in the interpretation of these outcomes, as some level of HS was observed even with active cooling.
